# Pre-treatment with a Xingnaojing preparation ameliorates sevoflurane-induced neuroapoptosis in the infant rat striatum

**DOI:** 10.3892/mmr.2014.2934

**Published:** 2014-11-13

**Authors:** ZHOU-JING YANG, YING-WEI WANG, CHANG-LIN LI, LI-QING MA, XUAN ZHAO

**Affiliations:** Department of Anesthesiology and Intensive Care Medicine, Xinhua Hospital, Shanghai Jiaotong University School of Medicine, Shanghai 200092, P.R. China

**Keywords:** chinese medicine, sevoflurane, apoptosis, signal transduction, protein kinase B signaling, striatum

## Abstract

Xingnaojing (XNJ), is a standardized Chinese herbal medicine product derived from An Gong Niu Huang Pill. It may be involved in neuroprotection in a number of neurological disorders. Exposure to anesthetic agents during the brain growth spurt may trigger widespread neuroapoptosis in the developing brain. Thus the present study aimed to identify whether there was a neuroprotective effect of XNJ on anesthesia-induced neuroapoptosis. Seven-day-old rats received treatment with 2.1% sevoflurane for 6 h. Rat pups were injected intraperitoneally with 1 or 10 ml/kg XNJ at 0.2, 24 and 48 h prior to sevoflurane exposure. The striata of neonatal rats were collected following administration of anesthesia. Western blotting and immunohistochemistry were used to analyze the expression of activated caspase 3, Bax and phosphorylated protein kinase B (p-AKT) in the striatum. It was found that activated caspase 3 and Bax expression were upregulated in the striatum following sevoflurane treatment. Preconditioning with XNJ attenuated the neuronal apoptosis induced by sevoflurane in a dose-dependent manner. Anesthesia reduced the expression of p-AKT (phosphorylated at sites Thr308 and Ser473) and phosphorylated extracellular-regulated protein kinase (p-ERK) in the striatum. Pre-treatment with XNJ reversed the reduction in p-AKT, but not p-ERK expression. These data suggest that XNJ has an antiapoptotic effect against sevoflurane-induced cell loss in the striatum. It thus holds promise as a safe and effective neuroprotective agent. The action of XNJ on p-AKT may make a significant contribution to its neuroprotective effect.

## Introduction

Sevoflurane is a widely-used volatile anesthetic for the induction and maintenance of general anesthesia, particularly in pediatric anesthesia, due to its rapid induction and the rapid recovery of patients. It has been reported that exposure of the neonatal nervous system to sevoflurane may lead to widespread neurodegeneration in a number of regions of the developing rat brain and that it may cause learning abnormalities in mice (1,2). The process of cell death triggered by sevoflurane exhibits all the classical ultrastructural characteristics of apoptosis (3–5) and is known to be regulated by the Bax-dependent intrinsic pathway, involving cytochrome *c* release from the mitochondria and activation of the caspase cascade (6–8). Currently there is no available prophylactic treatment to prevent anesthesia-induced neuroapoptosis.

An Gong Niu Huang Pill is a well-known traditional Chinese medicine used in the treatment of certain neurological disorders. It contains *Calculus bovis*, *Cornu bubali*, Cinnabar, *Curcuma aromatica*, *Scutellaria*, *Coptidis rhizoma* and *Fructus gardenia* (30 g of each), Moschus and Borneo camphor (7.5 g of each) and *Concha margaritifera* (15 g) (9). Xingnaojing (XNJ), an extract of An Gong Niu Huang Pill, predominantly contains Moschus (*Moschus berezovskii* F.; 7.5 g), *Radix curcumae* (*Curcuma wenyujin* Y.H. Chen & C. Ling, Zingiberaceae; 30 g), *Borneolum* (*Blumea balsamifera* DC, *Compositae*; 1 g) and *Fructus gardenia* (*Gardenia jasminoides* J. Ellis, Rubiaceae; 30 g) (10). XNJ is a herbal preparation of moschus, borneolum, *Fructus gardenia* and *Radix curcumae* and is primarily used in the treatment of neurological diseases, including brain ischemia, neurotoxic damage, cerebral hemorrhage, bacterial and viral meningitis and vascular dementia (10–12). It is unclear whether XNJ may protect against sevoflurane-induced neuronal apoptosis in the developing brain. Thus, the present study was designed to investigate the possible neuroprotective effects of XNJ, and to attempt to elucidate the underlying mechanisms of any such effects.

## Materials and methods

### Preparation and quality control of XNJ

XNJ was obtained from Wuxi Jiminkexin Shanhe Pharmaceutical Co., Ltd. (Wuxi, China) with the Chinese Food and Drug Administration number z32020563. It is registered by the Ministry of Public Health of China under the Chinese traditional patent formulation no. 17 (standard code, WS3-B-3353-98). The experiments were repeated in five different batches (batch numbers: 120313, 121101, 121205, 120506 and 120103). The result were highly similar with each batch. XNJ was prepared from four traditional Chinese medicines, including Moschus, Borneolum, *Radix curcumae* and *Fructus gardenia* (10). These herbs were identified by Professor Yuning Yan of Beijing University of Chinese Medicine (Beijing, China). Dried *Radix curcumae* and *Fructus gardenia* (~30) were dissolved into 1.5 liters of water. The solution was distilled, leaving 1 liter of liquid. Moschus (7.5 g) and 250 ml distilled water were added for further distillation and 1 liter of liquid was collected. Borneolum (1 g) with 8 g polysorbate 80 were added to the mixed solution. Finally, 8 g sodium chloride was added. The solution was filtered, sterilized and transferred into ampoules (13). In accordance with the corresponding quality control standard, XNJ should contain no less than 0.7 g/l Borneolum (molecular formula, C_10_H_18_O) (13).

The chemical fingerprint of XNJ has been identified in previous studies. The central active components of XNJ are muscone (PubChem CID, 10947), L-borneol (PubChem CID, 6850744), L-camphor (PubChem CID, 230921), isoborneol (PubChem CID, 6321405), curdione (PubChem CID, 6441391), germacrone (PubChem CID, 6436348), curcumin (PubChem CID, 969516) and geniposide (PubChem CID, 107848) (10,14–17).

### Animals

The study protocol was approved by the Animal Use and Care Committee for Research and Education of Shanghai Jiao Tong University (Shanghai, China). Sprague Dawley rats (Shanghai SLAC Laboratory Animal Co., Ltd, Shanghai, China) used in this study were maintained under a 12 h light/dark cycle (7am–7pm) with a room temperature of 22 ± 1°C. Food and water were available *ad libitum* for the lactating rats. Pups were divided into groups with approximately equal numbers of males and females for all experiments. The number of animals used and any suffering were minimized.

### Anesthesia treatment

On postnatal day seven (P_7_), rat pups were placed into a chamber and exposed to 2.1% sevoflurane for 6 h. The total gas flow was 1.5 l/min, using 70% O_2_ as a carrier. The oxygen and anesthetic agent fractions were measured using a gas analysis system (GE Healthcare Bio-Sciences, Pittsburgh, PA, USA). During exposure to the anesthetic, the chamber was maintained at 37 ± 1°C with an infrared heat lamp. Animals were sacrificed following 6 h of anesthesia.

### Pre-treatment strategies

**Figure 1:**
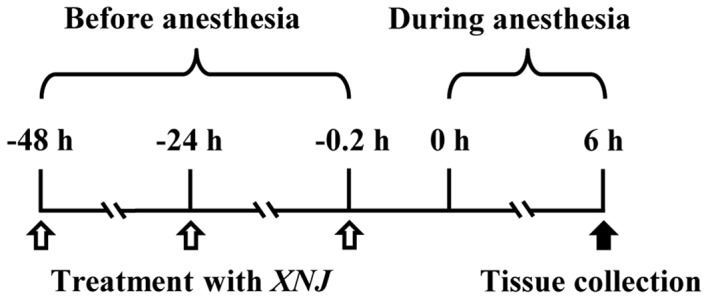
Schematic of XNJ pre-treatment and sevoflurane exposure. XNJ, xingnaojing.

Rat pups were injected intraperitoneally with 1 or 10 ml/kg XNJ (Henan New Century Pharmaceutical Co., Ltd., Henan, China) at 0.2, 24 and 48 h prior to anesthesia. Rat pups in the control group received an equal volume of saline (Fig. 1).

### Western blot analysis

Striatal tissues were homogenized in radioimmunoprecipitation assay lysis buffer, pH 7.4, containing 50 mM Tris-HCl, 150 mM NaCl, 1 mM EDTA, 1% NP-40, 0.25% sodium deoxycholate, 0.1% sodium dodecyl sulfate (SDS), a protease inhibitor cocktail (Thermo Fisher Scientific, Pittsburgh, PA, USA), and phosphatase inhibitors (10 mM Na_3_VO_4_, 10 mM NaF). Following homogenization, samples were centrifuged at 15294 xg for 10 min. Equal quantities of protein lysates were loaded onto a 10–15% SDS-polyacrylamide gel electrophoresis gel and transferred onto a 0.2 μm polyvinylidene difluoride membrane (EMD Millipore, Billerica, MA, USA). The membrane was blocked with 5% non-fat milk to reduce nonspecific binding, and immunobloted overnight using primary antibodies against Bax (1:2,000 dilution; Epitomics, Burlingame, CA, USA), activated caspase 3 (1:500 dilution), phosphorylated protein kinase B (p-AKT; at site Thr308; 1:1,000 dilution), p-AKT (at site Ser473; 1:1,000 dilution), AKT (1:5,000 dilution), phosphorylated extracellular-regulated protein kinase (p-ERK; 1:1,000 dilution), ERK (1:1,000 dilution), phosphorylated c-Jun N-terminal kinase (p-JNK; 1:5,000 dilution) and JNK (1:1,000 dilution; all Cell Signaling Technology Inc., Danvers, MA, USA). Anti β-actin antibody (1:200 dilution; Santa Cruz Biotechnology, Inc., Dallas, TX, USA) was used as a control to confirm equal loading. Immunocomplexes were visualized using an enhanced chemiluminescence-detection reagent (EMD Millipore). Band intensities were measured with Quantity One 4.4.0 software (Bio-Rad Laboratories, Hercules, CA, USA).

### Immunohistochemistry

The rats of sevo group and sevo plus pre-treatment with XNJ group were exposed to 2.1% sevoflurane for 6 h. Soon after they revived, the rats of all the groups, including the control group, XNJ group, sevo group and sevo plus pre-treatment with XNJ group, were anesthetized with sodium pentobarbital (60 mg/kg; Harbin Pharmaceutical Group Co., Ltd., Harbin, China) to minimize their suffering. The rats were sacrificed for immunohistochemistry analysis. The brains were perfusion-fixed with 4% paraformaldehyde and 0.1% picric acid in 0.1 M phosphate buffer (pH 7.4) followed by immersion fixation in the same mixture overnight. Subsequently, one hemisphere was kept in 20% sucrose in 0.1 M phosphate buffer for at least two days at 4°C. Sagittal sections (20 μm) were cut using a vibratome (Leica Microsystems GmBH, Wetzlar, Germany). Frozen tissue sections were incubated for 10 min with 3% H_2_O_2_ in 100% methanol to inactivate any endogenous peroxidase. Tissues were permeabilized and non-specific binding was blocked using 0.2% Triton X-100 and 5% heat-inactivated donkey serum in phosphate-buffered saline (PBS). Primary rabbit monoclonal antibodies against activated caspase 3 (Cell Signaling Technology Inc., 1:200) were diluted in 5% donkey serum/PBS and incubated overnight at 4°C. Specimens were incubated in biotinylated goat anti-rabbit IgG (diluted 1:500 in PBS containing 1% normal goat serum; Beyotime Institute of Biotechnology, Shanghai, China) for 3 h at room temperature. Sections were developed using the VECTASTAIN ABC reagent (Vector Laboratories, Inc., Burlingame, CA, USA) in PBS with 0.1% Tween-20 for 30 min. A DAB substrate kit (Vector Laboratories Inc., Burlingame, CA, USA) was used for immunohistochemical staining. Tissue sections were examined under a light microscope (Leica DM400B; Leica Microsystems GmBH). The number of activated caspase 3-positive neurons in the striatum of the developing rats was counted with Image J 1.48u software (National Institutes of Health, Bethesda, Maryland, USA).

### Statistical analysis

The experiments were performed with five different batches of XNJ and repeated at least three times. Statistical analyses were performed using SigmaPlot version 10.0 (Systat Software Inc., San Jose, CA, USA). All data are expressed as the mean ± standard error of the mean. Comparisons among multiple groups involved one-way analysis of variance plus Newman-Keul’s multiple comparison test. P<0.05 was considered to indicate a statistically significant difference.

## Results

### Dose-dependent effect of XNJ on the expression of activated caspase 3 in the striatum of the developing rat brain following sevoflurane treatment

**Figure 2:**
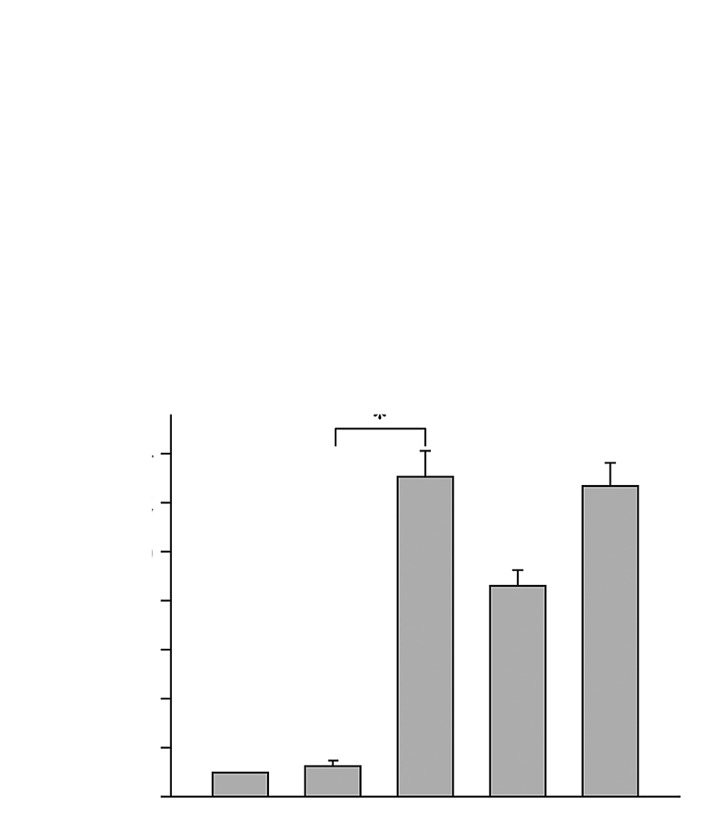
XNJ inhibition of caspase 3 activity. Upper panel: Representative western blot showing the expression of activated caspase 3 in the striatum of seven-day-old rats in: Lane 1, the control group; lane 2, the *XNJ* group; lane 3, the sevo group; lane 4, the sevo plus pre-treatment with 10 ml/kg *XNJ* group; and lane 5, the sevo plus pre-treatment with 1 ml/kg *XNJ* group. Lower panel: Results are compared with those of β-actin. Quantitative data are presented as the mean ± standard deviation (n=5).^*^P<0.05. XNJ, xingnaojing; sevo, sevoflurane.

Caspases are cysteine proteases that are involved in apoptosis. Increased expression and activation of caspase family members is known to contribute to neuronal death (18). Western blot analysis was performed to quantify the expression levels of activated caspase 3. Caspase 3 activity was significantly increased in the striatum of the developing rats following 6 h of sevoflurane treatment compared with the control group (Fig. 2). The increase of caspase 3 activity was partially inhibited by pre-treatment with 10 ml/kg XNJ (Fig. 2), but not by pre-treatment with the lower dose of 1 ml/kg (Fig. 2). Furthermore, treatment with XNJ alone did not increase the activity of caspase 3 in the striatum of neonatal rats (Fig. 2).

**Figure 3:**
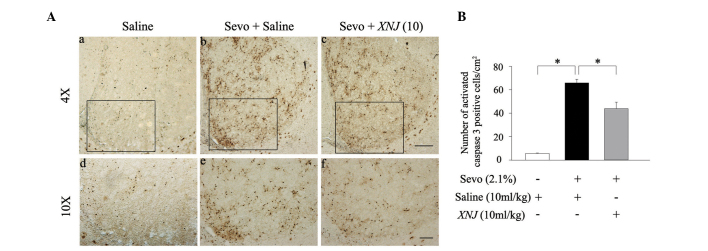
Effect of XNJ on the expression of activated caspase 3 in neonatal striatum. (A) Activated caspase 3-positive cells are stained brown and can be observed in the area surrounding the striatum. Low-magnification (4X) images show transverse hemisections from the striatum of seven-day-old rats in the (Aa) control group, (Ab) sevo group and (Ac) sevo pre-treatment with 10 ml/kg XNJ (scale bar, 10 mm). Enlarged images (10X) showed the boxed region of striatum in (Ad) the control group, (Ae) sevo group and (Af) sevo plus pre-treatment with 10 ml/kg XNJ (scale bar, 4 mm). (B) Average activated caspase 3 positive cells/cm^2^ in the striatum were counted. Quantitative data are presented as the mean ± standard deviation (n=5). ^*^P<0.05. XNJ, Xingnaojing; Sevo, sevoflurane.

The expression of activated caspase 3 following 6 h of sevoflurane treatment was also measured using immunohistochemistry. There were few activated caspase 3-positive neurons in the striatum of the developing rats that did not receive sevoflurane anesthesia (Fig. 3Aa and d). However, the number of activated caspase 3-positive neurons was significantly increased following 6 h of administration with sevoflurane (2.1%; Fig. 3Ab and e). Pre-treatment with 10 ml/kg XNJ reversed the sevoflurane-induced increase of activated caspase 3-positive neurons in the striatum of neonatal rats (Fig. 3Ac and f). The statistical data from this experiment concurred with the data from the western blot analysis. (Fig. 3B). These results show that sevoflurane-induces neuronal apoptosis in the striatum and that this effect may be counteracted by administration of XNJ.

### Effect of XNJ on sevoflurane-induced upregulation of apoptosis-related protein Bax levels in the striatum of rat pups

**Figure 4:**
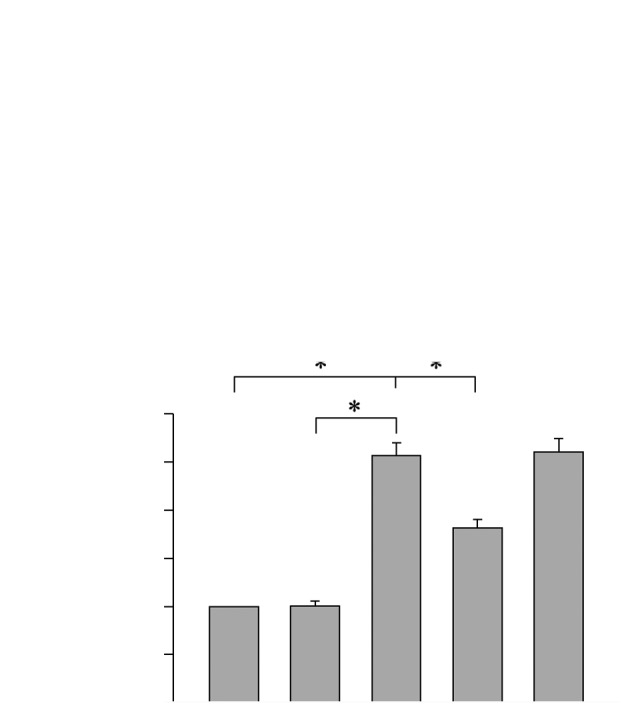
Effects of XNJ on sevo-induced upregulation of Bax protein in the developing rat brain striatum. Upper panel: Representative western blot showing the expression of Bax in the striatum of seven-day-old rats in: Lane 1, the control group; lane 2, the XNJ group; lane 3, the sevo group; lane 4, the sevo plus pre-treatment with 10 ml/kg XNJ group; and lane 5, the sevo plus pre-treatment with 1 ml/kg XNJ group. Lower panel: Quantitative data are presented as the mean ± standard deviation (n=5). ^*^P<0.05. XNJ, Xingnaojing; Sevo, sevoflurane.

The proapoptotic protein, Bax, localizes to the mitochondria in response to numerous apoptotic stimuli and leads to the release of cytochrome *c,* which further activates the caspase cascade (19). Therefore, the expression level of Bax was measured in the striatum of neonatal rats following 6 h of sevoflurane treatment in the absence and presence of 10 ml/kg XNJ. The expression of Bax in the striatum was upregulated following sevoflurane treatment compared with the control group, whilst pre-treatment with 10 ml/kg XNJ significantly reversed the sevoflurane-induced increase in Bax expression (Fig. 4). However, pre-treatment with 1 ml/kg XNJ had no significant protective effect on the sevoflurane-induced upregulation of the Bax protein (Fig. 4).

These results show that treatment with sevoflurane results in an increase in Bax expression, which may lead to neuroapoptosis. However, pre-treatment with XNJ significantly reduced this upregulation of Bax, which indicates a possible neuroprotective effect.

### Effect of XNJ and sevoflurane on Akt, ERK1/2 and JNK expression

**Figure 5:**
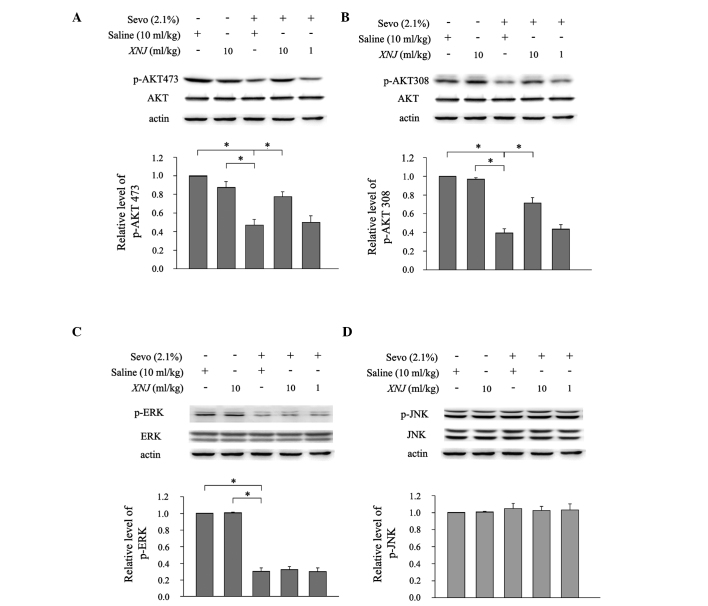
Effect of XNJ on the expression of p-AKT, p-ERK and p-JNK in the striatum following sevoflurane exposure. Upper panel, representative western blots; lower panel, quantitative data showing expression of (A) p-AKT (Ser473), (B) p-AKT (Thr308), (C) p-ERK and (D) p-JNK in the striatum of seven-day-old rats in: Lane 1, the control group; lane 2, the XNJ group; lane 3, the sevo group; lane 4, the sevo plus pre-treatment with 10 ml/kg XNJ group; and lane 5, the sevo plus pre-treatment with 1 ml/kg XNJ group. β-actin was used as a protein loading control. Quantitative data are presented as the mean ± standard deviation (n=5). ^*^P<0.05. XNJ, xingnaojing; Sevo, sevoflurane; p-AKT, phosphorylated protein kinase B; p-ERK, phosphorylated extracellular-regulated kinase; p-JNK, phosphorylated c-Jun N-terminal kinase.

It is reported that the AKT, ERK and JNK pathways are involved in the modulation of neuronal apoptosis (20). The present study aimed to investigate the effects of pre-treatment with XNJ on these three signaling cascades. Western blot analysis showed that sevoflurane significantly reduced the levels of p-AKT (Ser473; Fig. 5A) and p-AKT (Thr308; Fig. 5B) in the striatum of rat pups. Pre-treatment with 10 ml/kg XNJ reduced the suppressant effect of sevoflurane on p-AKT levels (Fig. 5A and B). XNJ at a dose of 1 ml/kg did not significantly affect the expression of p-AKT (Fig. 5A and B). Notably, sevoflurane anesthesia had no significant effect on the levels of the unphosphorylated AKT protein (Fig. 5A and B).

Sevoflurane anesthesia also reduced the expression of p-ERK in the striatum of neonatal brain (Fig. 5C), which was in accordance with a previous study (20). However, pre-treatment with 1 or 10 ml/kg XNJ did not counteract this decrease in p-ERK expression (Fig. 5C). In comparison to p-AKT and p-ERK, sevoflurane anesthesia had no inhibitory effect on the expression of p-JNK (Fig. 5D).

These results indicate that sevoflurane-induced neuronal apoptosis is modulated by the PI3K/AKT and ERK pathways. Furthermore, administration of 10 mg/kg XNJ modulated the effect of sevoflurane on the PI3K/AKT pathway, but not the ERK pathway.

## Discussion

Exposure to sevoflurane (2.1%) for 6 h significantly increased neuronal apoptosis in the striatum of P_7_ rat brain. It has been shown that there is no evidence of hypoxia in rat pups following treatment with sevoflurane at a concentration of 2.1% (21). The increased apoptosis detected in the present study was therefore predominantly a result of the anesthesia. The current study aimed to investigate the effect of pre-treatment with XNJ on sevoflurane-induced neuronal apoptosis in the neonatal rat brain. XNJ was administered at 0.2, 24 and 48 h prior to anesthesia. The results showed that pre-treatment with 10 ml/kg XNJ reversed in part the sevoflurane-induced neuronal apoptosis in the striatum of the neonatal rat brain.

It has been shown that neonatal exposure to sevoflurane may cause deficits in a number of aspects of cognitive function later in life, including maternal behavior, learning disabilities, social behaviors, adaptability changes and behavior in fear conditioning tests (2,22,23). The striatum is composed of functional sub-units that are part of cortico-striatal-thalamic circuits. Recent studies have focused on the contribution of striatal sub-regions to planning and decision making, declarative memory retrieval, learning of associations between stimulation, actions and rewards, selection among alternatives and modulation of motor behavior (24–26). Thus, neuroprotection of the striatum against volatile anesthetics is an important area of research, particularly in pediatric anesthesia.

*An Gong Niu Huang Pill* is a well-known traditional Chinese medicine. In traditional medicine, it is believed to cure the illness, known as ‘shutting syndrome’, in which the patient is thought to lose consciousness as a result of too much sputum, fire or blot in the meridian. The herbs treat the patient by keeping these factors away from the brain, something that is termed the ‘Xingnaokaiqiao’ method (9). A previous study showed that *An Gong Niu Huang Pill* promotes the recovery of neonatal hypoxic-ischemic encephalopathy and answers the safety for the newborn babies (27). XNJ, an extract of *An Gong Niu Huang Pill*, has similar clinical applications. A previous study investigated the pharmacokinetics of muscone, borneol and geniposide, the main components of XNJ, following injection into the tail veins of rats. The time taken to reach their maximum concentrations in the brain was ~5 min for muscone (28), 1 min for borneol (29) and 1 min for geniposide (30). The time taken to reach their maximum concentrations in the brain when administered nasally was ~3.4 min for borneol (29) and 3 min for geniposide (30). The action of XNJ is rapid and relatively long-lasting in the brain (31). The duration of XNJ pre-treatment in the present study was based on these findings.

XNJ has a direct effect on the central nervous system as it is able to cross the blood brain barrier (32). It has been shown to be effective in a number of neurological diseases, including cerebrovascular disorders, central nervous system infections, cerebral injury, alcoholism and seizure disorders (17,33–35). XNJ enhances learning and memory, and facilitates cognitive function in older animals following ketamine treatment (36,37). Furthermore, XNJ may exert an antiapoptotic effect (11). These studies led to the hypothesis that XNJ may inhibit apoptosis triggered by sevoflurane in the developing rat brain.

The mechanism of neuronal apoptosis has been well investigated. Bax and activated caspase 3 are likely to be involved in neuronal apoptosis during synaptogenesis (38,39). Caspase 3 is a cysteine protease that is involved in mediating cell apoptosis. It results in cell shrinkage, DNA degradation and the formation of apoptotic bodies (40). The proapoptotic protein, Bax, localizes to the mitochondria in response to numerous apoptotic stimuli and leads to the release of cytochrome *c,* which further activates the caspase cascade. Therefore Bax is important in a number of apoptotic signaling pathways (41). It has been shown that anesthetic exposure may expedite physiological apoptosis by elevating the expression of Bax and activated caspase 3 (38). The results from the present study supported this finding and showed that pre-conditioning with XNJ reduced these changes in rat striatum.

To further investigate the effects of XNJ, the activity of the MAPK and PI3K/AKT pathways, which have potential neuroprotective actions, was investigated. AKT, ERK and JNK kinases are known to be involved in regulating diversified physiological and pathological processes in the brain, including neuronal survival, apoptosis, proliferation, differentiation, development and synaptic plasticity (42). High levels of growth factors, particularly those that activate PI3K, may favor the survival of certain cell types with the aid of AKT (43). The PI3K/AKT signaling pathway, which triggers the phosphorylation of the serine/threonine kinase AKT, protects cells from apoptosis by increasing the activity of proteins that promote cell survival and suppressing caspases involved in promoting apoptosis (44,45). AKT is primarily phosphorylated at two sites: Thr308 and Ser473 (46). In the present study, sevoflurane treatment inhibited the expression of p-AKT (Thr308) and p-AKT (Ser473) in the striatum of rat neonatal brain, although it had no significant effect on the expression of the AKT protein. These results were consistent with those from previous studies (20,38). It was found that pre-treatment with 10 mg/kg XNJ prior to anesthesia reduced the inhibitory effect of sevoflurane on the expression of p-AKT (at Ser473 and Thr308), but did not alter the expression of the AKT protein in the striatum of rat neonatal brain. Although sevoflurane inhibited the phosphorylation of ERK, pre-treatment with 10 mg/kg XNJ had no significant effect on ERK phosphorylation in the striatum. It was also demonstrated that sevoflurane anesthesia did not alter the activity of JNK in the striatum. Notably, treatment with 10 mg/kg XNJ alone had no significant effect on the activity of AKT, ERK or JNK in the striatum of neonatal rat brain, which implies it may be safe to use in neonatal rodents.

**Figure 6:**
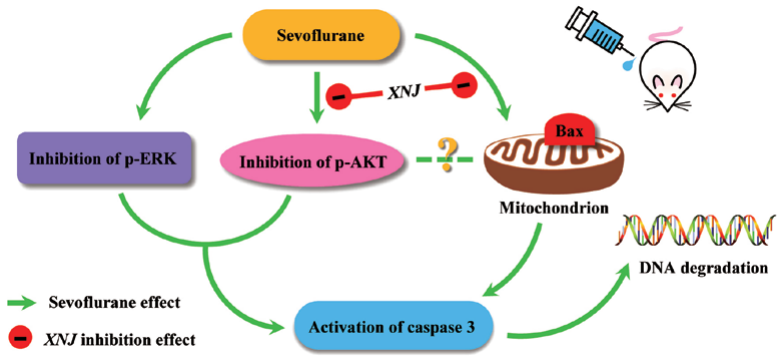
Schematic showing XNJ-induced protection against neurodegeneration in the striatum of developing rats caused by sevoflurane. XNJ, xingnaojing.

It has been reported that a combination of two or more kinase systems may be required in order to trigger apoptosis. AKT suppression leads to apoptosis when the ERK pathway is suppressed simultaneously (20,47). It is therefore likely that sevoflurane-induced neuroapoptosis results from suppression of the p-AKT and p-ERK pathways. In addition, the current study suggested that there may be a point at which XNJ interferes with the apoptotic signaling pathway. Pre-treatment with XNJ reversed the sevoflurane-induced reduction in p-AKT, but not p-ERK expression. Thus phosphorylation of AKT may be the step at which XNJ exerts its antiapoptotic and neuroprotective effects. To the best of our knowledge, there are no previous studies showing that the effect of XNJ on p-AKT expression protects against drug-induced neuroapoptosis. Elucidation of the exact mechanism requires further investigation. Furthermore, activation of AKT results in phosphorylation of the proapoptotic protein, Bax. This interferes with the stability of Bax, abrogates its proapoptotic function and aids in promoting cell survival (48). The present study found that pre-conditioning with XNJ reduced neuronal apoptosis via regulation of Bax, but did not confirm the association between p-AKT and Bax, which requires further investigation (Fig. 6).

To date, a number of approaches to combat anesthesia-induced neonatal brain injury have been addressed in numerous animal models, including pre-conditioning with pyruvate, lithium, nicotinamide, N-stearoyl-l-tyrosine and nucleosome assembly protein (20,38,49,50). Although these drugs have yielded favorable responses, the adverse effects of these compounds on the brains of infants remain unclear (20). In traditional Chinese medicine, XNJ is believed to act on the patients by the principle of ‘jun-chen-zuo-shi’, on a systematic level, in which the active components interact with each other, enhancing the efficacy and reducing the side effects of this compound. It has traditionally been used in China and no significant adverse effects are known. The current study showed the neuroprotective effect of sevoflurane-induced neuronal apoptosis in neonatal rat brain, indicating that XNJ may be effective against anesthesia-induced neurotoxicity in the infant brain. However, the underlying mechanisms of this effect remain unclear and further investigation is required in order to demonstrate its safety and efficacy in human infants.

In conclusion, pre-treatment with 10 ml/kg XNJ may have a neuroprotective effect against sevoflurane-induced neuronal apoptosis in the striatum of neonatal rat brain. The effect of XNJ on the phosphorylation of AKT may contribute to this neuroprotective effect.
